# Au Bipyramids as NIR-II Contrast Agents for In Vivo Plant Imaging

**DOI:** 10.1021/acsami.5c08908

**Published:** 2025-08-21

**Authors:** Luis D. B. Manuel, Debarati Basu, Mary Beth Rollins, Vinoin Devpaul Vincely, Pelham Keahey, Martin Villiger, Carolyn L. Bayer, Paul F. South, Kevin M. McPeak

**Affiliations:** † Gordon and Mary Cain Department of Chemical Engineering, 5779 Louisiana State University, Baton Rouge, Louisiana 70803, United States; ‡ Department of Biological Sciences, Louisiana State University, Baton Rouge, Louisiana 70803, United States; § Department of Plant Pathology and Crop Physiology, Louisiana State University College of Agriculture, Baton Rouge, Louisiana 70803, United States; ∥ Department of Biomedical Engineering, 5783 Tulane University, New Orleans, Louisiana 70118, United States; ⊥ 1811 Harvard Medical School, Boston, Massachusetts 02115, United States; # Wellman Center for Photomedicine, Massachusetts General Hospital, Boston, Massachusetts 02114, United States

**Keywords:** plants, cytotoxicity, gold bipyramids, plasmonic nanoparticles, NIR-II, photoacoustic imaging, optical coherence tomography, spatiotemporal imaging

## Abstract

In vivo plant imaging is crucial for understanding plant biology and the influence of external factors on plant health. While exogenous contrast agents are widely used in animal bioimaging to enhance contrast, track flows, and provide molecular specificity, their application in plant tissue remains challenging and underexplored. Herein, we highlight the shortcomings of contrast agents in optical coherence tomography of plant tissue while demonstrating the successful use of Au bipyramids (AuBPs) as effective NIR-II contrast agents in photoacoustic imaging, enabling spatiotemporal flow tracking in live Buttercrunch lettuce over 5 days. Furthermore, rigorous plant health studies showed no adverse effects of the AuBPs on the physiological properties of Buttercrunch lettuce 7 days postinfiltration. The use of AuBPs as contrast agents in plant imaging, combined with the versatility of their surface chemistry, opens the possibilities for studies with molecular specificity.

## Graphical abstract

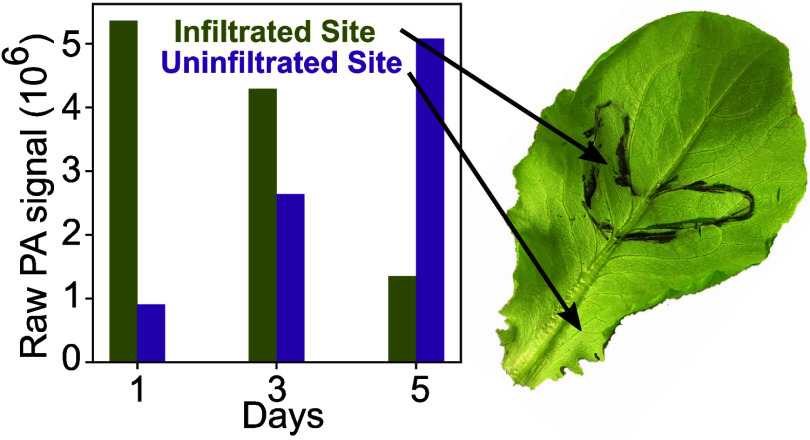

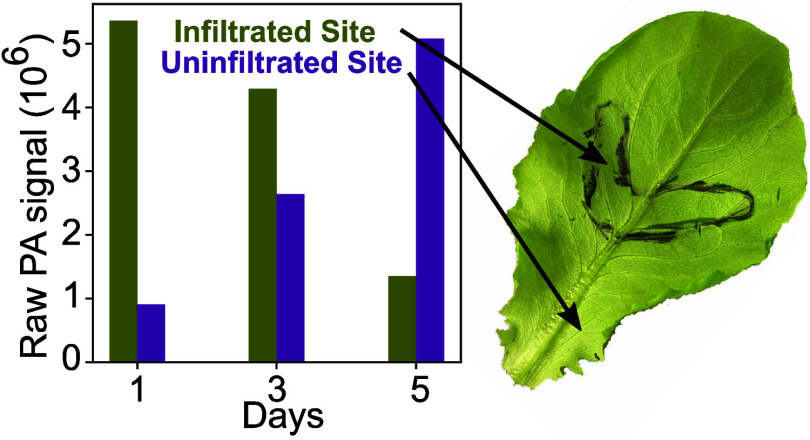

## Introduction

Plasmonic nanostructures are revolutionizing biomedical applications, with gold bipyramids (AuBPs) emerging as a particularly promising innovation.^1^ AuBPs offer a remarkable combination of features—sharp, tunable plasmonic resonances, exceptional biocompatibility, and versatility in functionalization—making them ideal for groundbreaking advancements in nanomedicine.^1^ AuBPs hold significant promise for applications requiring precise control of the light–matter interactions. Their elongated, sharp tips enhance plasmonic properties, resulting in stronger local electric fields, larger optical cross sections, narrower bandwidths, and greater refractive index sensitivity than most Au nanostructures.^1^ Additionally, AuBP plasmon resonances are highly tunable across both near-infrared (NIR) biological windows, NIR-I (650–950 nm) and -II (1000–1750 nm).^1^ Due to superior light penetration, these windows are essential for in vivo biomedical applications.

Consequently, the use of AuBPs has been successfully extended to many in vivo studies. Yuan et al. demonstrated the ability of AuBPs to provide distinct optical labels during optical coherence tomography (OCT) imaging of the lymphatic system in live mice.^2^ Similarly, Keahey et al. leveraged polarization-sensitive and spectroscopic OCT to differentiate multiple AuBPs from surrounding tissues and blood vessels in live mice, thus achieving greater imaging sensitivity.^3^ The multifunctionality of AuBPs is illustrated by Feng et al., who integrated surface-enhanced Raman scattering (SERS) detection with targeted photothermal therapy using bioconjugated AuBPs, and Liu et al., who achieved photoacoustic signal detection and effective photothermal treatment in tumor-bearing mice by employing polydopamine-coated AuBPs.^4,5^ Xu et al. demonstrated the use of AuBPs for in vivo CT imaging and photothermal therapy in mice, further showcasing their versatility in multimodal imaging and therapeutic applications.^6^

Despite the progress of AuBPs in nanomedicine, to the best of our knowledge, there are no applications of AuBPs in plant imaging. Plants present unique challenges for bioimaging techniques due to their natural light-trapping efficiency, which inherently limits the detection of outgoing optical signals.^7–9^ Furthermore, light absorption by pigments, such as chlorophyll, and scattering caused by structural components like cell walls and rough surfaces can prevent light from penetrating the intended target within the plant.^7,9^ These challenges are not as pronounced in human tissue.^9^ The distinct challenges of bioimaging in plant and human tissues are illustrated in Figure 1. Figure 1a and b highlight the differences in structure and refractive index contrast between plant and human tissues. The inner structure of the plant leaf tissue comprises stacked mesophyll cells and air spaces. Meanwhile, human tissue has fewer structures that interact with light by comparison. Accompanying optical properties of both tissues are shown in Figure 1c and d. Figure 1c measures the absorption and scattering of a Buttercrunch lettuce leaf. As shown in Figure 1c, plant tissue exhibits high absorption and low scattering of visible light but low absorption and high scattering in the NIR. Figure 1d plots absorption and scattering data in the human fatty tissue measured by Haugen et al.^10^ In contrast, Figure 1d demonstrates that the human tissue scatters significantly less than plants in the NIR but absorbs more. The significant light scattering in plant leaves results from the difference in refractive indices between mesophyll cells and the air spaces within the plant.^11^ Conversely, the variance in the refractive index is not to the same extent in the human tissue. The high scattering cross-section in the plant tissue is a significant challenge for live plant imaging across various modalities.^12,13^ Introducing AuBPs as exogenous contrast agents may offer a viable solution to overcoming plant imaging challenges.

**Figure 1:**
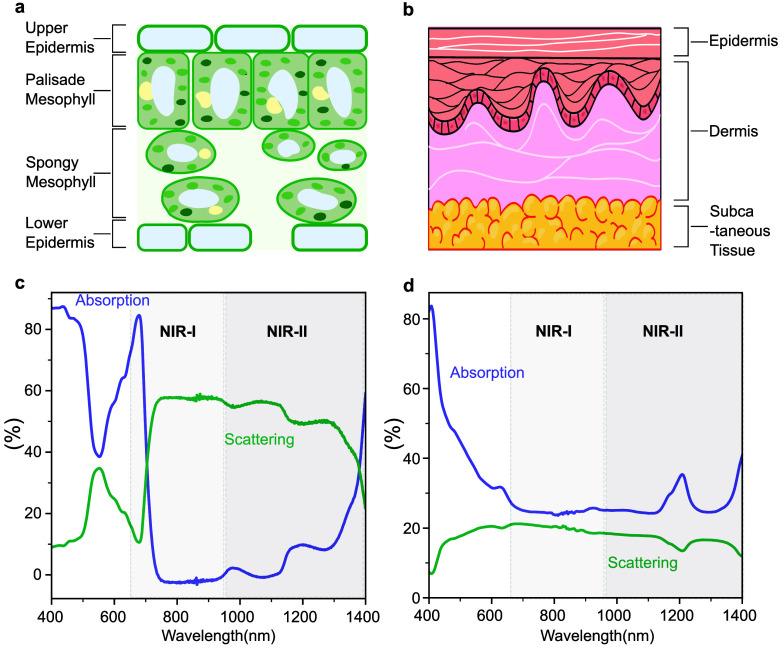
Challenges in optical bioimaging. Schematic representation of key tissue components influencing light extinction in the (a) plant tissue and (b) human tissue. Comparison of absorption and scattering properties in the (c) plant tissue and (d) human tissue,^10^ highlighting their distinct optical behaviors in the visible and NIR wavelengths.

Exogenous markers have been widely used in fluorescence-based plant imaging techniques^14–16^ such as confocal microscopy, which operates in the visible wavelength regime.^17^ However, the use of NIR-active exogenous contrast agents in plant imaging remains underexplored. While several imaging techniques could be suitable for NIR-active contrast agents, OCT and photoacoustic imaging (PAI) stand out as particularly promising.^18–21^ OCT utilizes the interference of coherent light to generate high-resolution images,^18^ while PAI combines the advantages of optical and ultrasound imaging by converting absorbed light into ultrasound waves via the thermoelastic effect.^19^ Both techniques have seen a surge in plant imaging applications without exogenous markers, in contrast to their widespread use in animal imaging, where such markers are common.

Over the past two decades, OCT has gained popularity in plant imaging, with studies demonstrating its potential for visualizing intact plant structures at the cellular level. For instance, Sapozhnikova et al. used the OCT to visualize individual plant cells, highlighting its utility in plant studies. Chow et al. employed OCT to detect viral infections in orchid plants.^22^ Additional studies have used OCT for pathogen diagnosis and plant growth monitoring.^23–25^ Similarly, PAI has been explored in plant research. Suzuki et al. used PAI to assess rice grain quality by evaluating PA signals corresponding to different water content levels.^26^ Khodakovskaya et al. introduced carbon nanotubes (CNTs) into tomatoes and utilized PAI to study cellular interactions, concluding that CNTs induced gene expression mutations.^27^ Other studies have leveraged PAI to analyze plant components and track metal content in plants.^28,29^ Despite these advancements, there remain few studies using nanoparticle-based exogenous contrast agents in plant imaging and, to the best of our knowledge, none implementing NIR-active exogenous contrast agents. This lack of nanoparticle-based plant imaging studies is exacerbated by the challenges and poor fundamental understanding of nanoparticle transport in the plant tissue.^30^ To address this, we aim to introduce and characterize NIR-active AuBP contrast agents in the OCT and PAI imaging techniques.

Herein, we demonstrate that gold bipyramids (AuBPs) are promising contrast agents for near-infrared (NIR) imaging in live plant systems. We selected vegetables as our test subjects because, as high-value crops, they play a critical role in human nutrition and global food security. Although vegetables represent a smaller share of global crop production compared to cereals, their productivity, which can be up to four times higher, has made them a central focus of agricultural research and development.^31^

Among vegetable crops, lettuce (*Lactuca sativa*), a member of the *Asteraceae* family, stands out as a widely cultivated leafy green with a substantial global economic value. Lettuce is a suitable model for molecular biology studies due to the availability of a high-quality reference genome and well-established transformation protocols.^32^ Furthermore, it has been extensively used to investigate how variations in light intensity, quality, and duration influence plant phenotype and physiological responses.^33^

In this study, we employed syringe infiltration to introduce AuBPs into Buttercrunch lettuce leaves. The large and accessible leaf surface of lettuce makes it particularly well-suited for evaluating the impact of nanoparticles on plant health. While conventional model plants are often selected for their well-characterized genomes and ease of genetic manipulation, we deliberately chose *L. sativa* over *Arabidopsis thaliana* and *Nicotiana benthamiana* due to its higher sensitivity to both abiotic and biotic stress, making it a more responsive system for detecting subtle physiological changes.

To the best of our knowledge, this is the first investigation into the physiological effects of AuBPs on plants and the first use of exogenous contrast agents for NIR-II imaging in live plant tissue. Using optical coherence tomography (OCT) and photoacoustic imaging (PAI), we performed NIR-II imaging of live Buttercrunch lettuce leaves enhanced with AuBPs. Our results demonstrate successful spatiotemporal tracking of AuBPs in plant tissues over several days. Additionally, postinfiltration plant health assessments revealed no adverse effects from the AuBPs, confirming their biocompatibility in this context.

## Results and Discussion

### Infiltration of AuBPs into Buttercrunch Lettuce

Following the seed-mediated crystal growth method by Chateau et al.,^34^ we synthesized AuBPs with an average length of 258 nm and a width of 45 nm, exhibiting peak resonance in the NIR-II window for infiltration into Buttercrunch lettuce leaves. Infiltration was performed using the syringe-infiltration method described by Wroblewski et al.,^35^ which involves applying pressure with a needleless syringe against the abaxial side of the leaf lamina (Video S1). We injected AuBP suspensions at concentrations ranging from 35 to 80 mg/L. We confirmed successful infiltration by performing elemental analysis using inductively coupled plasma optical emission spectroscopy (ICP-OES) to detect the presence of Au in the leaves, as shown in Table S1 of the Supporting Information. For comparison, we mock-infiltrated leaves with water (without AuBPs) to rule out infiltration-related stress. Hereafter, we use the terms “mock-infiltrated” and “uninfiltrated” interchangeably for simplicity, while “AuBP-infiltrated” will be referred to simply as “infiltrated”.

The selected AuBP concentrations were based on previous studies indicating that higher concentrations of Au nanoparticles (AuNPs) in plants increase the likelihood of adverse effects.^36–38^ Figure 2a presents examples of infiltrated and uninfiltrated leaves. Further details on the synthesis, properties, and plant infiltration of AuBPs are provided in Figures S1 through S3 of the Supporting Information. The following section discusses the impact of AuBPs on plant health.

**Figure 2:**
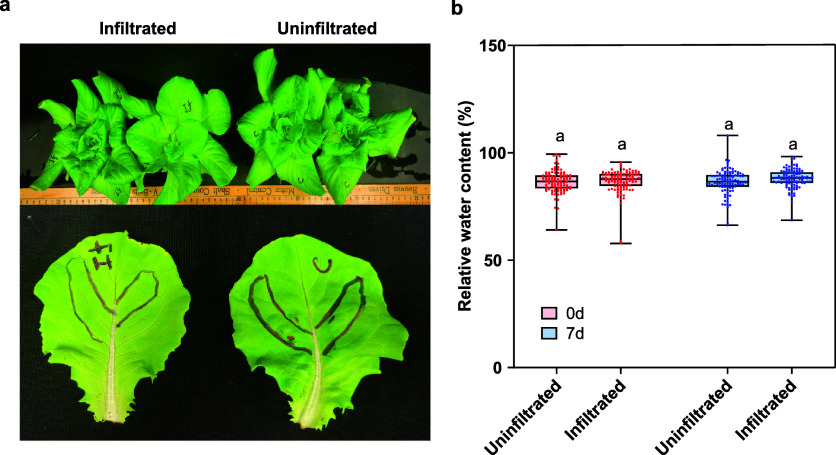
Phenotypical and relative water content comparison of AuBP infiltrated and uninfiltrated plants. (a) Representative lettuce plant and leaf image with 7 days post AuBP infiltration and uninfiltrated. (Left) infiltrated plants, (right) uninfiltrated plants. The black area marked indicates an area of infiltration. (Top panel) Five- to six-week-old lettuce plants. (Bottom panel) Infiltrated and uninfiltrated leaves. (b) Relative water content (%) measured at indicated time points representing the transpiration rate for uninfiltrated and infiltrated leaves. Nine leaves per treatment and time point were measured. Data was pooled from three trials.

### Impact of AuBP Treatment on Plant Health

Previous studies have shown that AuNP administration in nanomedicine can trigger unintended immunological responses, such as cytokine imbalances in mammalian hosts.^39,40^ Similarly, several studies have reported that AuNPs can negatively affect plant growth and development to varying degrees.^37,38^ To address potential biocompatibility concerns related to AuBPs and their surface ligands, we investigated whether foliar infiltration of lettuce plants affects the overall plant health. Nanoparticles are known to aggregate, and such aggregation can congest stomata on the leaf surface or obstruct vascular tissues.^41^ These blockages may interfere with gas exchange through the stomata or disrupt water and nutrient transport, thereby impairing key physiological processes, such as transpiration and photosynthesis. To assess these potential effects, we compared relative water content (RWC), as a proxy for plant transpiration, along with several photosynthetic parameters between uninfiltrated and infiltrated lettuce plants.^42,43^ This evaluation was conducted to ensure that no adverse physiological or immunological responses were induced by AuBP treatment.

The first indication of the absence of unintended immunological responses appears in Figure 2a, where infiltrated leaves exhibit no phenotypic changes in coloration compared to uninfiltrated leaves 7 days postinfiltration. Additional evidence comes from the RWC comparison results. Leaf RWC, shown in Figure 2b, reflects the balance between water supply to the leaf tissue and transpiration rate, serving as an indicator of plant water status, influenced by transport.^44^ Feichtmeier et al. previously demonstrated that higher concentrations of AuNPs (≥100 mg/L) can hinder water transport in plants.^36^ However, as shown in Figure 2b, no discernible differences in RWC were observed between uninfiltrated and infiltrated plants at 0 and 7 days postinfiltration. This result further validates our decision to use AuBP concentrations below 100 mg/L to mitigate potential adverse effects.

We also compared net photosynthesis, stomatal conductance, intercellular CO_2_ levels, and fluorescence measurements between uninfiltrated and infiltrated plants, as shown in Figure 3. Several studies suggest that AuNP infiltration may impair photosynthesis.^45,46^ To assess photosynthetic performance, we conducted gas-exchange measurements, examining net photosynthesis at low (100 ppm), ambient (400 ppm), and high (2250 ppm) atmospheric CO_2_ concentrations. Figure 3a shows that infiltrated leaves exhibited similar trends in net photosynthesis compared with uninfiltrated leaves. Likewise, Figure 3b indicates no detectable differences in intercellular CO_2_ concentrations between uninfiltrated and infiltrated lettuce leaves.

**Figure 3:**
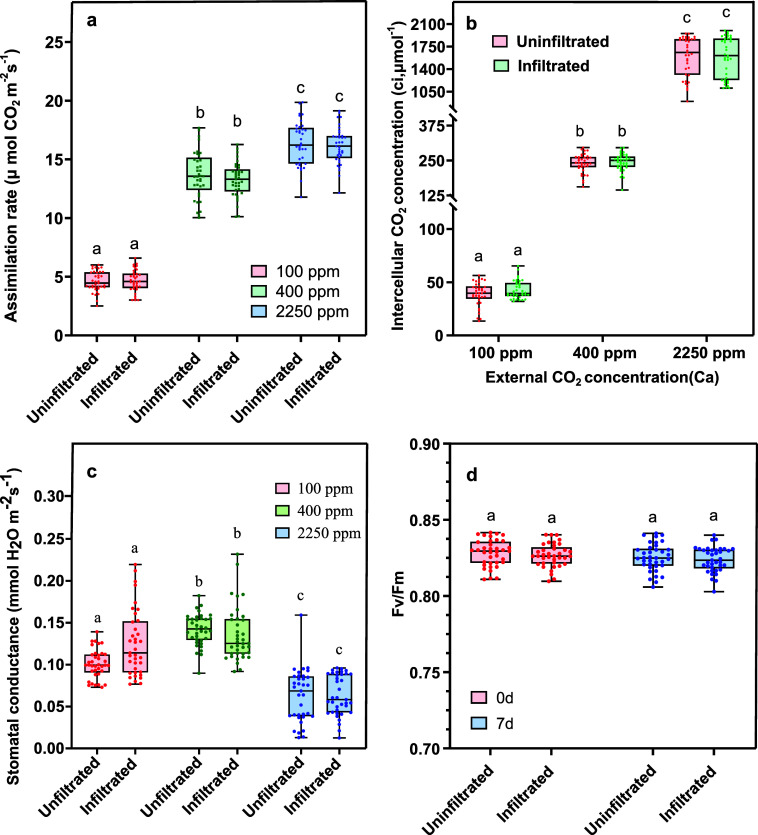
Effects of AuBPs on plant health were assessed by two-way ANOVA followed by a Tukey HSD post hoc test, *P* <0.05. Error bars represent standard deviation. (a) Assimilation rate measured with an infrared gas analyzer at a PAR of 1500 μmol photons m^–2^ s^–1^ representing photosynthetic efficiency rates determined at three CO_2_ concentrations 100, 400, and 2250 ppm. (b) Intercellular CO_2_ concentration (*C*_i_, μmol^–1^) at varying external CO_2_ concentrations (*C*_a_) in ppm. (c) Stomatal conductance (mmol H_2_O m^–2^ s^–1^) in uninfiltrated and infiltrated lettuce leaves. (d) Fv/Fm as a measure of quantum efficiency of photosystem II at 0 and 7 days postinfiltration. These results show that AuBPs cause no discernible health impairment to the lettuce plants in 7 days.

Additionally, we monitored stomatal conductance (H_2_O m^–2^ s^–1^) in uninfiltrated and infiltrated lettuce leaves at low (100 ppm), ambient (400 ppm), and high (2250 ppm) atmospheric CO_2_ concentrations. Since CO_2_ primarily enters through stomata, Figure 3c demonstrates that AuBP infiltration did not alter stomatal conductance at any of the tested CO_2_ concentrations. Lastly, we measured chlorophyll fluorescence parameters, as efficient utilization of incident light is critical for photosynthesis. Specifically, Figure 3d presents the variable fluorescence (Fv) to maximum fluorescence (Fm) ratio in uninfiltrated and infiltrated leaves. Similar to the other measured parameters, the Fv:Fm ratio showed no discernible differences between the two groups.

These results suggest that syringe infiltration of AuBPs at 80 mg/L did not negatively impact the physiology of lettuce plants within 7 days post-treatment, as assessed by RWC and photosynthetic parameters. As shown in Supporting Information, we also conducted similar assessments of the effect of AuBP infiltration on soybean plants, which yielded the same type of results. The results observed in soybean plants support the generalization that AuBPs do not cause adverse effects in other plant species. With this assurance of no adverse effects, we proceeded to conduct imaging studies using AuBPs as contrast agents.

### Optical Coherence Tomography and Photoacoustic Imaging

Our first imaging study was OCT on uninfiltrated and infiltrated leaves using the experimental setup depicted in Figure 4a, where the coverslip and ultrasound gel provide an index of refraction match to the leaf. Figure 4b presents a photograph of the experimental setup including an imaged leaf. Figure 4c shows the en-face view of the imaging area, represented by the red box in Figure 4b. Figures 4d–g display the resulting OCT images: Figure 4d and f show representative intensity tomograms at different magnifications, while Figure 4e and g present the corresponding degree-of-polarization (DOP) tomograms. Notably, the degree of polarization provides an additional source of contrast, leveraging the random orientation of the nanoparticles to induce light decoherence.^47^ Additionally, we computed 2D histograms illustrating the relationship between SNR and DOP, and we analyzed them across large areas of leaves with and without injected AuBPs, focusing on regions away from the veins. As shown in Figure 4h (with AuBPs) and **4i** (without AuBPs), the histograms are highly similar, with no evident increase in depolarization in the AuBP-infiltrated leaf.

**Figure 4:**
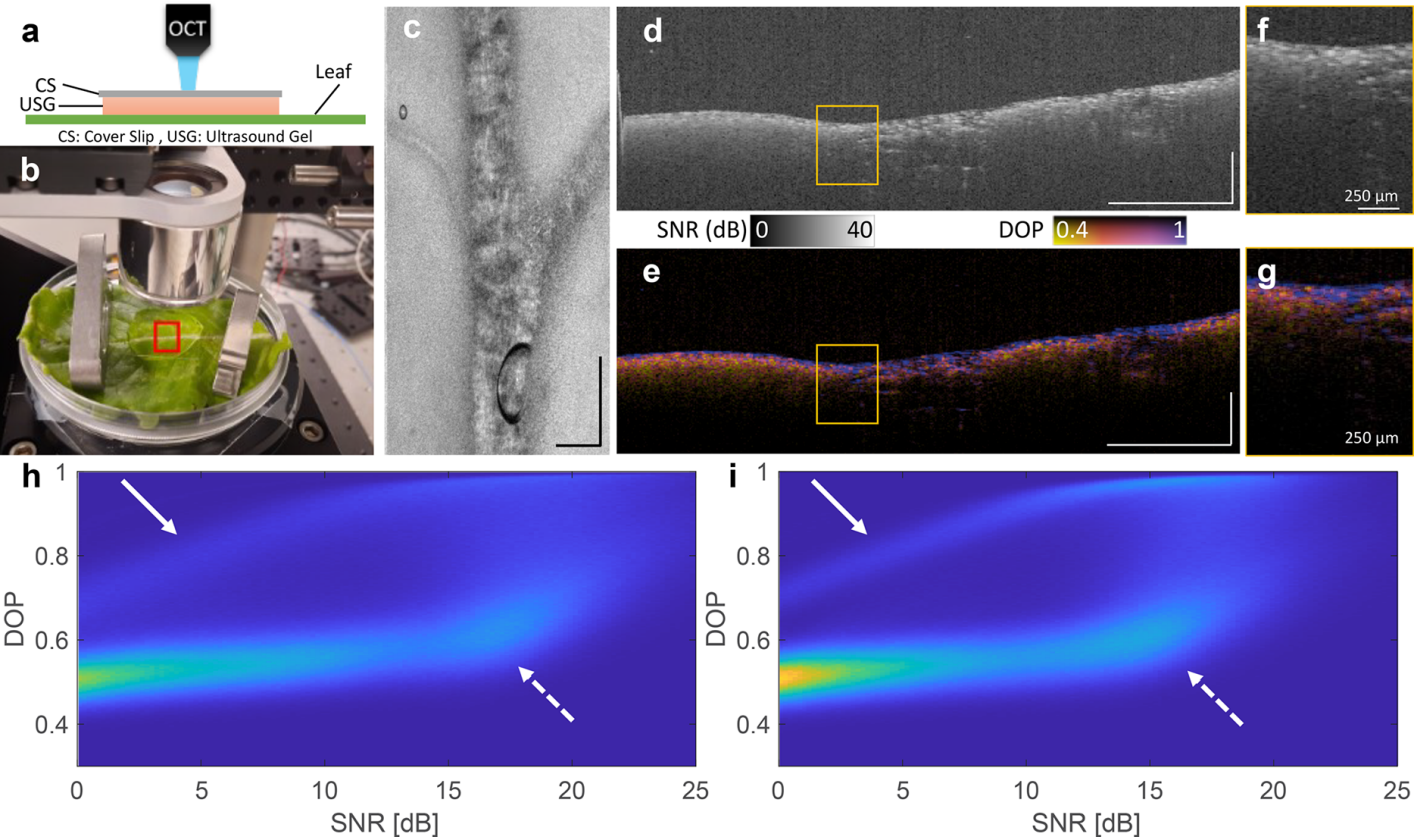
OCT imaging of the lettuce leaf structure. (a) Diagram of the experimental setup. (b) Photograph of leaf imaging. (c) En-face view of the 5 × 5 mm imaging area (red box in panel b). (d) Intensity tomogram of a representative cross-section of the leaf, SNR (dB) denotes signal-to-noise ratio in decibels. (e) DOP tomogram of the same cross-section. (f,g) Respective zoomed view of the intensity and DOP tomogram. Scale bars = 1 mm unless noted. (h,i) 2D histogram of SNR and DOP where the white arrow denotes noise-induced depolarization and dashed arrow denotes scattering-induced depolarization indicating little difference with (h) with AuBP’s presence and (i) without AuBP’s presence.

The resulting OCT imaging of infiltrated and uninfiltrated leaves revealed no significant differences, likely due to the substantial light scattering in the NIR spectrum of Buttercrunch lettuce, as shown in Figure 1c. The limited benefit of introducing exogenous agents for OCT imaging in plants aligns with challenges observed in other optical-based techniques. The primary difficulty stemmed from the significant multiple light scattering produced by the endogenous plant tissue, which made it challenging to distinguish depolarization changes caused by AuBPs from the background scattering. However, similar approaches have previously allowed for better differentiation than pure intensity measurements in animal tissues.^3^ In other scattering-based techniques, we similarly expect the background noise from the plant to overshadow the signal from the contrast agent.

Moving away from scattering-based techniques, we conducted PAI measurements on infiltrated leaves and tracked the movement of AuBPs from the infiltration site to distant uninfiltrated locations over a 5 day period. Figure 5a presents a schematic of the experimental setup, where the infiltrated leaf is embedded under an ultrasound gel layer, which facilitates the transport of the acoustic signal to a detector positioned perpendicular to the light source. In Figure 5b, we show the corresponding raw photoacoustic signals for both infiltrated and uninfiltrated sites with the respective imaging areas highlighted in Figure 5c. Figure 5d displays PA images showing a gradual decrease in the pixel intensity at the infiltration site, while Figure 5e shows a corresponding increase at the uninfiltrated site. The simultaneous decline in the PA signal at the infiltration site and the increase at the uninfiltrated site confirm the movement of AuBPs, as evidenced by the contrast quality in the corresponding PA images. A larger area showing the leaf boundary, the anatomy of interest, can be seen more clearly in the Supporting Information (Figure S5), including the corresponding ultrasound images.

**Figure 5:**
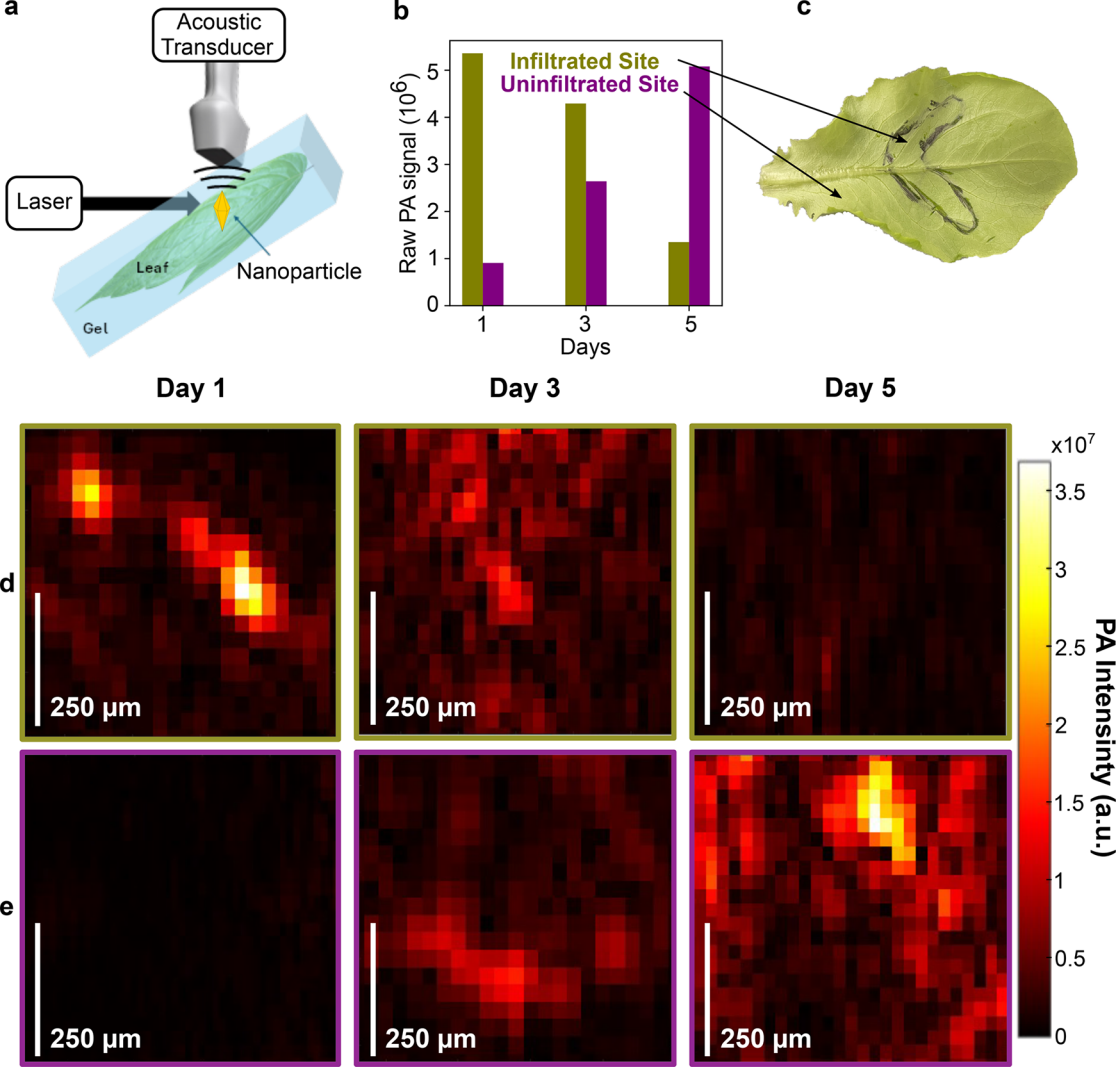
Setup and results of PAI for AuBPs in lettuce leaves. (a) PAI experimental setup scheme highlighting perpendicular light input to the acoustic sensor; (b) resulting raw PA signals detected; (c) plant image indicating infiltrated and uninfiltrated sites with corresponding signals over multiple days; (d) PA images of the infiltrated site over several days; (e) PA images of the uninfiltrated site over several days. The intensity scale bar of the images corresponds to the maximum signal amplitude of the received RF signal on a linear scale. A unique ROI around the imaged leaf region was defined, and the pixels within were averaged for further analysis.

These results confirm the movement of AuBPs within the plant, highlighting the potential for molecular imaging and marking the first use of NIR-II exogenous contrast agents in plants. The low absorption signals from Buttercrunch plants, as shown in Figure 1c, contribute minimal background noise, as opposed to the results from the OCT imaging results. Therefore, this technique demonstrates promise for molecular imaging in plants, with the next steps focused on identifying suitable ligands and molecules of interest.

## Conclusion

We compared in vivo the OCT and PAI of Buttercrunch lettuce leaves in the NIR-II using AuBP contrast agents. Significant background noise severely hindered OCT results. However, PAI provided superior results, successfully tracking AuBP movement for 5 days in live Buttercrunch lettuce leaves and establishing the potential for molecular PAI in plants. A reasonable conclusion is that other scattering-based techniques will face the same disadvantages as OCT. Therefore, techniques not affected by background noise from endogenous scattering, which can overshadow the contrast agent signal, are preferred. Background optical scattering does not undermine PAI due to its use of the thermoelastic effect from absorbed light. Other absorption-based imaging techniques could offer similar advantages. Plant health studies conducted at the same AuBP imaging concentration showed no adverse effects on lettuce plant physiology within 7 days post-treatment. These findings demonstrate that AuBP is a strong candidate for further PAI studies, advancing the molecular imaging potential in plants.

## Methods

### Materials

Cetyltrimethylammonium chloride (CTAC), octadecylamine hydrochloride (OHCl), nitric acid (HNO_3_), gold­(III) chloride trihydrate (HAuCl_4_), sodium borohydride (NaBH_4_), sodium hydroxide (NaOH), citric acid, cetyltrimethylammonium bromide (CTAB), silver nitrate (AgNO_3_), 8-hydroxyquinoline (HQL), tetramethylguanidine (TMG), and catechol were purchased from Sigma-Aldrich and Acros organics.

### AuBP Synthesis

We synthesized AuBPs following the seed-mediated crystal method by Chateau et al.^34^ This procedure involves three main steps. In the first step, pentatwinned Au seed crystals are prepared. In the second step, these seeds are overgrown to larger sizes and purified by centrifugation. Finally, in the third step, the purified seeds are used to grow AuBPs of the desired size.

To prepare the seed crystals, 1.65 g of CTAC and 55 mg of OHCl were dissolved in 77.95 mL of H_2_O and sonicated in a bath ultrasonicator at 40 °C until they were completely uniform. After cooling the solution to room temperature, 720 μL of HNO_3_ and 800 μL of 25 mM HAuCl_4_ were added while stirring at 1000 rpm. Stirring continued for 5 min before 1 mL of a freshly prepared 50 mM NaBH_4_/NaOH mixture was added rapidly to the vortex. One min after the NaBH_4_/NaOH addition, 100 μL of 0.1 M citric acid was introduced to form the seeds.

The seeds were then heated for 3.5 h to improve the yield. Seed overgrowth was carried out by adding a solution composed of 38 mL of 140 mM CTAB, 4.4 mL of 25 mM HAuCl_4_, 800 μL of 40 mM AgNO_3_, 1.1 mL of 0.1 M NaOH, and 2.4 mL of 0.4 M HQL in ethanol. This overgrowth step was conducted at 48 °C for 1 h. The resulting colloid was concentrated 12-fold by centrifugation at 10,000 RCF for 15 min and stored for future use.

Final bipyramidal growth was initiated by combining the overgrown seeds with a growth solution. The growth solution was prepared by mixing 1 mL of 100 mM HAuCl_4_, 3 mL of 137 mM CTAB, and 180 μL of 0.8 M tetramethylguanidine (TMG). After 2 min of stirring, 80 μL of AgNO_3_ and 650 μL of catechol (prepared in 90% H_2_O and 10% ethanol by volume) were added. Growth was performed by mixing 1.5 mL of 137 mM CTAB, 20 μL of 40 mM AgNO_3_, and 35 μL of the overgrown seed solution, stirred for 2 min at 58 °C, followed by the addition of the growth solution. The reaction was allowed to proceed for 20 min. Refer to Figure S1 in the Supporting Information for the properties of the synthesized AuBPs.

The desired reaction temperatures were achieved by submerging the reaction beakers in a mineral oil bath placed on a hot plate equipped with a thermocouple for temperature control (IKA RET Control Visc.)

### Plant Growth

Lettuce (*L. sativa* var. capitata), more specifically, Nancy, a Buttercrunch variety, was used in this study. Seeds were procured from a commercial supplier (Johnny’s Selected Seeds, Winslow, ME, USA) for experiments involving plant physiology and PAI. In contrast, for OCT imaging, Buttercrunch plants from Clegg’s nursery were used (Clegg’s Nursery, LLC, Baton Rouge, LA, USA). For consistency, fully expanded inner leaves were used for infiltration studies. Plants were grown in individual 4′ × 3.5′ pots containing soil mix (Sun Gro Horticulture Distribution Inc., MA) in Percival growth chamber LED 75L1 (Percival Scientific; Perry, IA, USA) maintained at 22 °C. The light intensity was maintained at 150 μmol m^–2^ s^–1^. Plants were grown using a 12 h-light/12 h-dark cycle under light conditions. Five-week-old to six-week-old plants were used for infiltrations.

### Plant Infiltration of AuBPs

Syringe infiltration was used to introduce AuBPs into lettuce leaves, as described by Wroblewski et al.^35^ with minor modifications (Video S1). Briefly, for plant physiology experiments, either 1 mL of water or 1 mL of 80 mg/L AuBPs conjugated with PEG resuspended in water was infiltrated. This involved infusion of the bipyramid suspension by applying pressure against the abaxial side of a leaf lamina with a 1 mL syringe without a needle. For monitoring the effect of infiltration at 0 days, plants were immediately assessed using LI-COR (LI-COR Inc., Lincoln, NE, USA) and again monitored 7 days postinfiltration. Each trial consisted of three plants infiltrated on three leaves (a total of nine leaves) on at least two different dates. After infiltration, the plants were maintained under the same conditions in which they were grown. We provided examples of infiltrated plants and rougher leaves to be avoided in Figures S2 and S3 of the Supporting Information, respectively.

### Elemental Analysis

We conducted an elemental analysis using a method similar to that of Avellan et al.,^48^ with modifications. First, the infiltrated plants were dried at 80 °C for 48 h. They were then digested overnight at room temperature in a 2:1 ratio of concentrated HNO_3_ to 30% H_2_O_2_, followed by heating at 95 °C for 30 min. After cooling, HCl was added to achieve a 2:1 HNO_3_/HCl ratio, and the samples were heated again at 95 °C for another 30 min. After second cooling, the samples were diluted in aqua regia for further digestion. Finally, the samples were diluted 10-fold, and ICP-OES analysis was conducted using a PerkinElmer Optima 8000 Optical Emission Spectrometer.

### Measurement of Relative Water Content

The measurement of relative water content (RWC) was performed using the method adapted from Barrs and Weatherley RWC was measured using leaves infiltrated with water and Au-BP suspension at 0 and 7 d.^43^ Briefly, three leaf discs (0.3 cm diameter) from each leaf were collected and weighed together to determine the fresh weight (FW). Next, three-leaf discs were transferred to a 12-well microplate containing 2 mL of distilled water. The leaf discs were hydrated overnight to attain full turgidity. Subsequently, excess water was removed by gently dabbing the leaf discs on blotting paper, and samples were weighed to determine the maximum turgid weight (TW). Finally, dry weight (DW) was determined by drying the leaf discs for 48 h in a gravity convection oven (VWR, Radnor, PA) at 65 °C. RWC is calculated using the formula RWC was calculated as RWC (%) = [(FW – DW)/(TW – DW)] × 100.

### Combined Gas Exchange and Chlorophyll Fluorescence Measurements

A Portable Photosynthesis System LI-6800 (LI-COR Inc., Lincoln, NE, USA) was used. Measurements were performed on the fully expanded leaf infiltrated with either water or AuBPs 7 days postinfiltration to measure gas exchange and chlorophyll fluorescence. Measurements were performed at a CO_2_ concentration of (low) 100 ppm, (ambient) 400 ppm, and (high) 2250 ppm, the photosynthetic photon flux density of 1200 μmol m^–2^ s^–1^, leaf temperature of 22 °C–23 °C, and relative humidity of 50%. Other chamber conditions were as follows: fan speed, 10,000 rpm; flow rate to the sample cell, 500 μmol air/s; and overpressure, 0.1 kPa. The leaf chamber fluorometer (6800-01A, area 2 cm^2^) was used for all of the measurements involving LI-6800.

For chlorophyll, PAM fluorescence measurements (Fv/Fm) were done using 6800-01A multiphase flash fluorometers (LI-COR Inc., Lincoln, NE, USA). Three leaves per plant were selected and dark-adapted for 3 h by wrapping the leaves with aluminum foil. Next, dark-adapted leaves were illuminated with weak modulated measuring beams 1 Hz, 2.5 μmol photons m^–2^ s^–1^, to obtain the initial fluorescence (*F*_0_). Finally, saturating white light pulses of 10,000 μmol photons, m^–2^ s^–1^, were applied for 0.8 s to ensure maximum fluorescence emissions (Fm), from which the variable to maximum chlorophyll fluorescence ratio, Fv/Fm = [(Fm – F_0_)/Fm], was calculated.

### OCT Imaging

We used a portable PS-OCT system built using a commercial Santec (Hackensack, NJ) swept-source with a central wavelength of 1310 nm, sweep range of 100 nm, and 50 kHz repetition rate. The incident power on the sample was 16.1 mW with a system sensitivity of 104 dB. All images were acquired with a LSM03 lens (ThorLabs, Newton, NJ), which provides an approximate spot size of 20 μm.

Employing the Stokes formalism, the degree of polarization (DOP) was averaged over the input polarization states *p* and the 3 spectral bands *n*:

$$DOP=\frac{1}{2N}\sum_{p=1}^{2}\sum_{n=1}^{N}\frac{\sqrt{{\left\langle Q_{p,n}\right\rangle }^{2}+{\left\langle U_{p,n}\right\rangle }^{2}+{\left\langle V_{p,n}\right\rangle }^{2}}}{\left\langle I_{p,n}\right\rangle }$$

Here, a Stokes vector is composed of the four components *I*, *Q*, *U*, and *V*, and ⟨.⟩ indicates lateral filtering with a Gaussian filter spanning 6 A-lines. DOP is always in the range of 1 (completely polarized) to 0 (completely depolarized). Afterward, DOP and intensity values were combined in a custom hue (DOP) saturation (intensity) color scheme shown in multiple figures.

### Photoacoustic Imaging

Photoacoustic (PA) imaging was performed using a custom integrated pulsed optical parametric oscillator (OPO) laser (Phocus HE, Opotek Inc., Carlsbad, CA, USA) and an open-architecture data acquisition system (Vantage 256, Verasonics Inc., Kirkland, WA, USA) coupled to a 6 MHz linear array transducer (L11-5v, Verasonics Inc., Kirkland, WA, USA). All PA images were acquired at a 1200 nm wavelength with an optical energy of 15 mJ (a Gaussian beam with a 5 mm diameter). The optical energy density incident on the leaf surface was measured using a power meter (Ophir Technologies, West North Logan, UT, USA) with an average pulse-to-pulse variation in laser output recorded under 5%. Multiple Buttercrunch leaves at different (1, 3, and 5) days post injection of nanopyramid were imaged. Given an air beam was used for illumination, the leaf had to be oriented at an angle (∼45°) to ensure the site of injection was adequately illuminated and that the transducer was positioned with this site in its field of view. The incline was obtained by encasing the leaf in a thick layer of ultrasound gel (Figure 5a). The transducer was visually positioned to image the site of injection, with the border of the leaf clearly visible on the ultrasound image (refer to supplemental Figure S5). All image acquisition was controlled through a MATLAB 2022a (Mathworks Inc., CA, USA) graphical interface, with image reconstruction performed using a proprietary Verasonics reconstruction algorithm. A region of interest (ROI) was drawn around the leaf border in the B-mode images, aligning the lateral dimensions of the ROI to the area illuminated by the air beam. The pixels within each ROI in the coaligned PA image were averaged for comparison (Figure 5d and e).

## Supplementary Material





## Notes

Video of a plant injection procedure example showing the method in which a bipyramid suspension is injected by applying pressure to the abaxial side of a leaf lamina with a 1 mL syringe without a needle, causing no damage to the leaf (MP4)
AuBP properties, plant infiltration examples, ICP results, and additional photoacoustic imaging results (PDF)
