# Supplementary material for: Au Bipyramids as NIR-II Contrast Agents for In Vivo Plant Imaging
Source: ACS Appl Mater Interfaces. 2025 Aug 21;17(35):49183–92. doi: 10.1021/acsami.5c08908 (PMC12412101; doi:10.1021/acsami.5c08908)
Supplement: Supplementary file 2 [file am5c08908_si_002.pdf]

Supporting Information for:

Au Bipyramids as NIR-II Contrast Agents for *In Vivo* Plant Imaging

Luis D. B. Manuel[1], *Debarati Basu*[2], *Mary Beth Rollins*[3], *Vinoin Devpaul Vincely*[4], *Pelham Keahey*[5,6], *Martin Villiger*[5,6], *Carolyn L. Bayer*[4], *Paul F. South*[2], *Kevin M. McPeak*[1]*

[1]Gordon and Mary Cain Department of Chemical Engineering, Louisiana State University, Baton Rouge, LA 70803, USA. [2]Department of Biological Sciences, Louisiana State University, LA 70803, USA. [3]Department of Plant Pathology and Crop Physiology, Louisiana State University College of Agriculture, Baton Rouge, LA 70803, USA. [4]Department of Biomedical Engineering, Tulane University, New Orleans, LA 70118, USA. [5]Harvard Medical School, Boston, MA, USA. [6]Wellman Center for Photomedicine, Massachusetts General Hospital, Boston, MA, USA.

Corresponding Author E-mail*: kmcpeak@lsu.edu

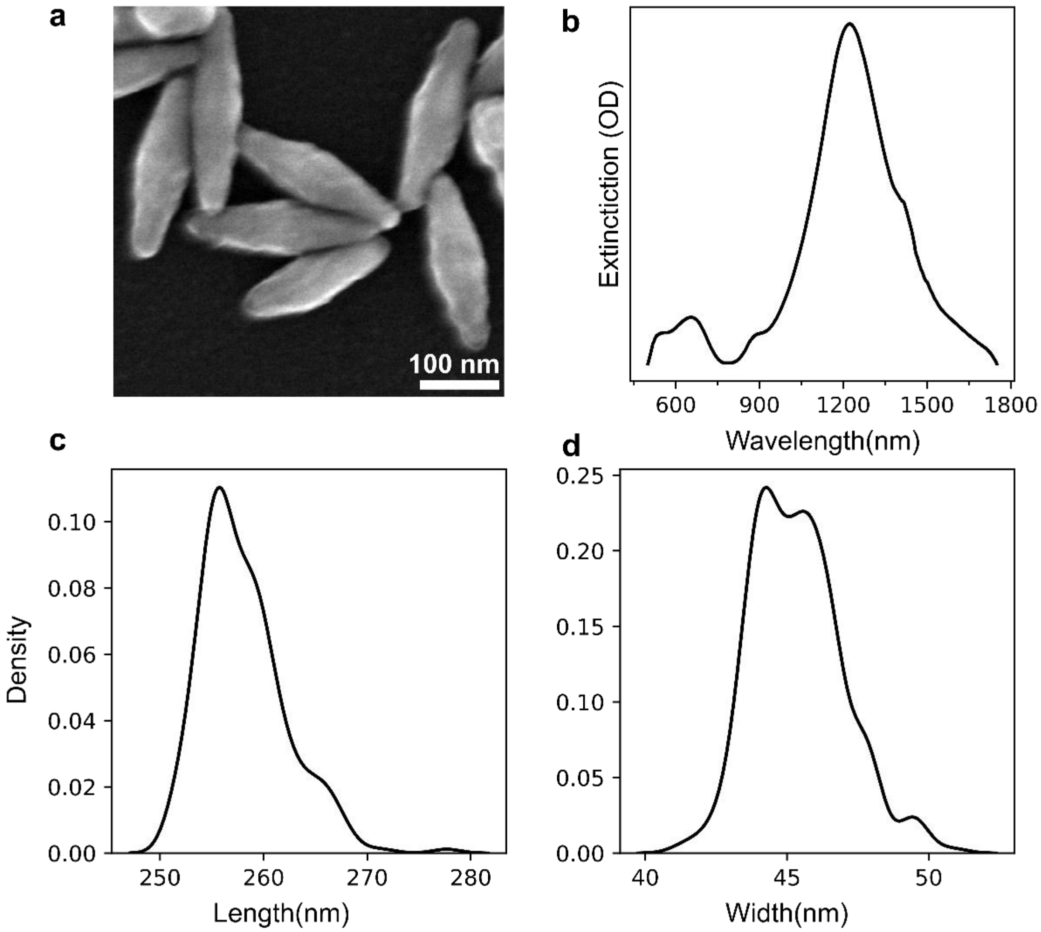

Figure S1. Properties of Au Bipyramids. (a) Scanning electron micrographs; (b) Extinction spectrum showing NIR-II resonance; (c, d) Size distribution of AuBPs: (c) length, with an average of 258 nm and a standard deviation of 4 nm; (d) width, with an average of 45 nm and a standard deviation of 1.6 nm.

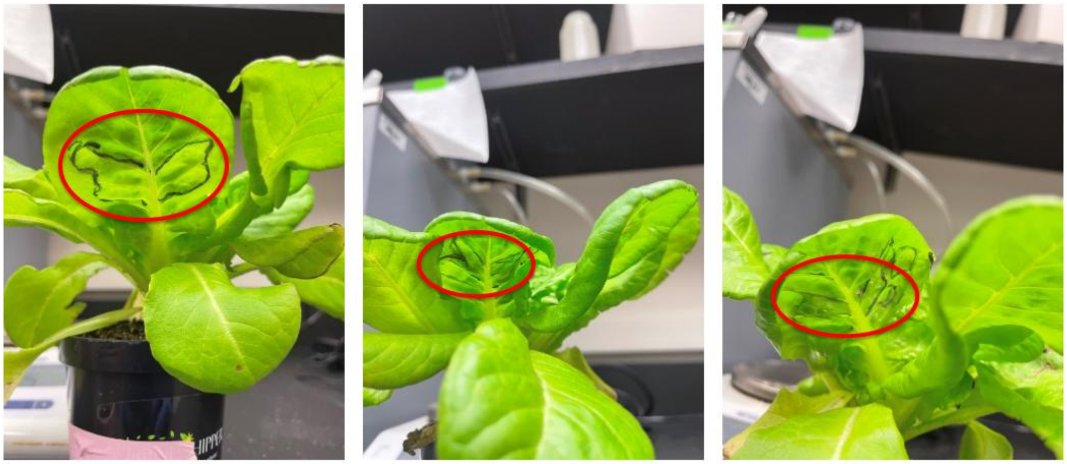

Figure S2. Examples of infiltration sites on Buttercrunch lettuce leaves. The marked areas show the initial region where the infiltrated suspension spreads

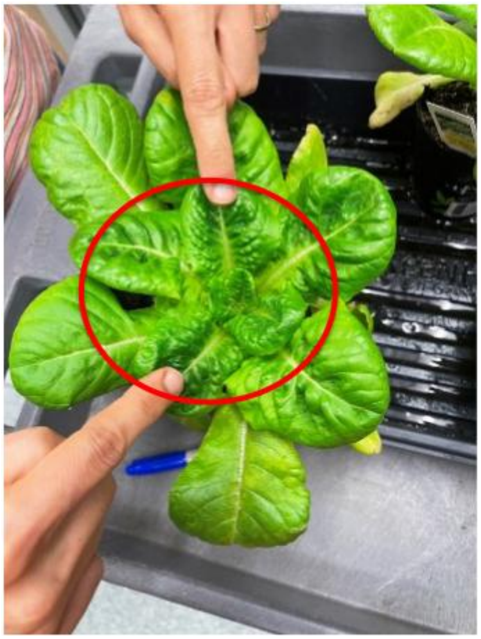

Figure S3. Examples of sites not ideal for infiltration for Buttercrunch lettuce leaves. Younger leaves can be significantly challenging to infiltrate due to rougher surfaces

Table S1. ICP-OES results showing Au BP presence beyond the infiltration site

Site	Au (µg/g)
Above Infiltration Spot	8.67
Infiltration Spot	5.78
Below Infiltration Spot	1.82

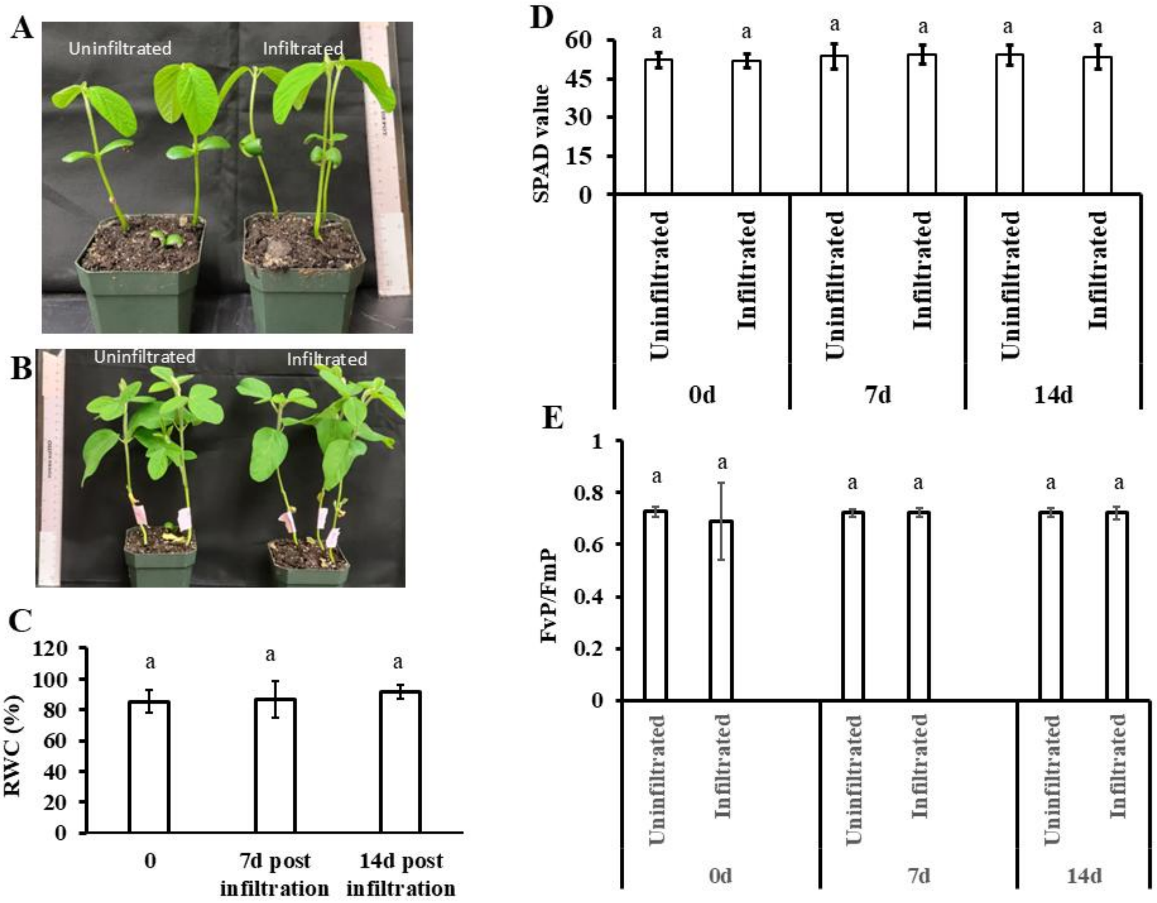

Figure S4. Effect of treatment of AuBPs on soybean health. **A-B.** Representative soybean plants at 7 days (A) and 14 days (B) post-infiltration, comparing mock-treated (uninfiltrated) and AuBs\P-infiltrated plants. **C.** RWC (%), realtive water content measured at indicated time points

representing transpiration rate of uninfiltrated and AuBPs-infiltrated soybean leaves. Six leaves per treatment and time point were measured. **D.** SPAD values representing total chlorophyll content, measured from uninfiltrated (water-infiltrated) and AuBPs-infiltrated leaves using a handheld MultispeQ V2.0 device connected to the PhotosynQ platform. **E.** FvP/FmP or maximum photochemical efficiency of photosystem II measured from uninfiltrated and AuBPs-infiltrated leaves using a handheld MultispeQ V2.0 device connected to the PhotosynQ platform. **C-E**. Values are means ± SD. The significant differences between uninfiltrated and infiltrated were tested using two-way ANOVA followed by a Tukey HSD post hoc test, $P < 0.05$. Same letters in the bar graph indicate that those samples are not significantly different.

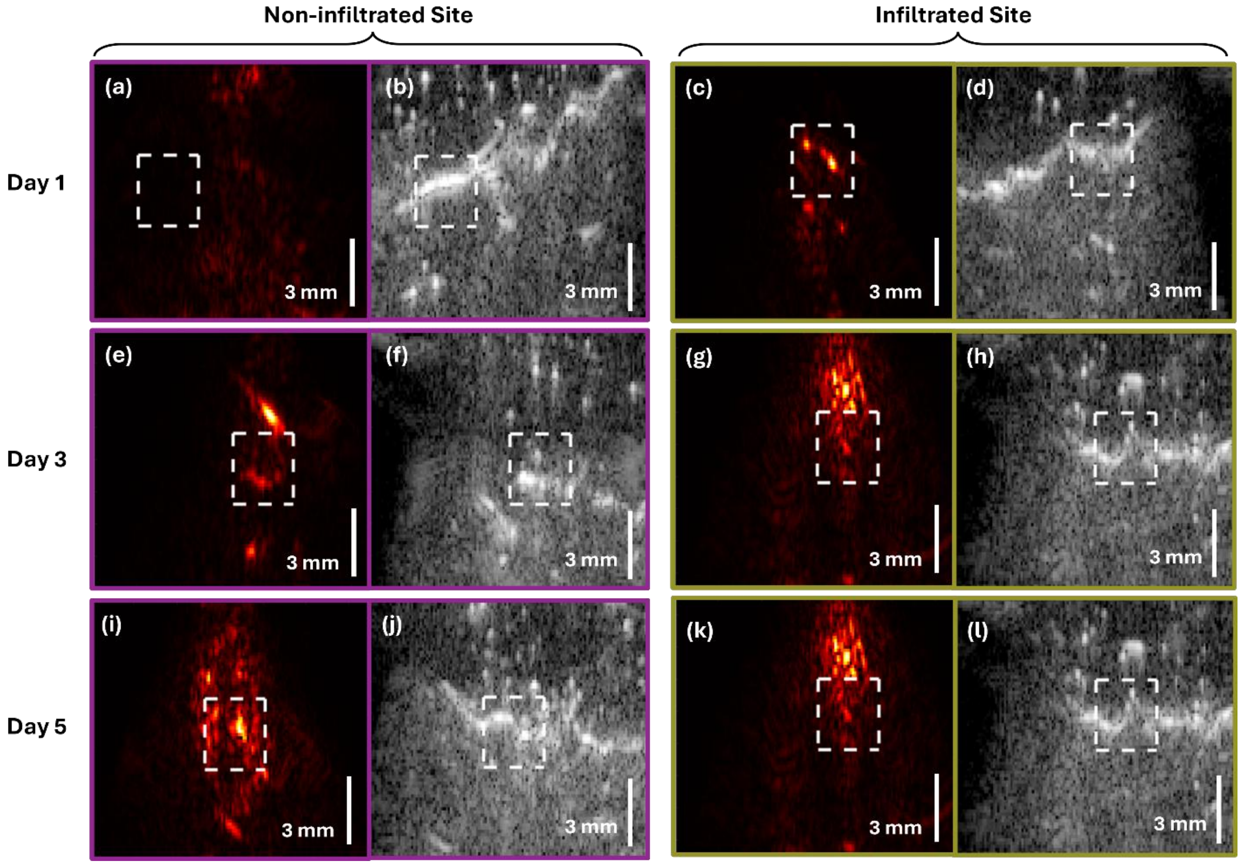

Figure S5. Photoacoustic imaging of leaf injected with bipyramids. PA (a, c, e, g, i, k) and B-mode (b, d, f, h, j, l) images of lettuce leaves injected with bipyramids for 1 (a-d), 3 (e-h) and 5 (i-l) days. Figures a, b, e, f, i, j are images of the uninfiltrated site while c, d, g, h, k, l are images of the infiltrated site (i.e. cite of injection). Each ROI (indicated with white dashed lines) was defined using the corresponding B-mode image, clearly showing the edge of each leaf (bright line), as a reference.

Video S1. A plant injection procedure example showing the method in which a bipyramid suspension is injected by applying pressure to the abaxial side of a leaf lamina with a 1 mL syringe without a needle, causing no damage to the leaf.
